# Effect of *Helicobacter pylori *on metabolic syndrome parameters in diabetic patients

**Published:** 2016-12

**Authors:** Jamshid Vafaeimanesh, Mohammad Bagherzadeh, Atefeh Mirzaei, Mahmoud Parham, Mohsen Norouzinia, Reza Vafaee

**Affiliations:** 1 *Gastrointestinal and Liver Disease Research Center, Iran University of Medical Science, Tehran, Iran*; 2 *Qom Gastroenterology and Hepatlogy Research Center, Qom University of Medical Sciences, Qom, Iran*; 3 *General Proctitioner, Qom University of Medical Sciences, Qom, Iran*; 4 *Endocrinologist, Qom Gastroenterology and Hepatlogy Research Center, Qom University of Medical Sciences, Qom, Iran*; 5 *Gastroenterology and Liver Diseases Research Center, Research Institute for Gastroenterology and Liver Diseases, Shahid Beheshti University of Medical Sciences, Tehran, Iran*; 6 *Proteomics Research Center, Shahid Beheshti University of Medical Sciences, Tehran, Iran*; 7 *Safety Promotion and Injury Prevention Research Center, Shahid Beheshti University of medical sciences, Tehran, Iran*

**Keywords:** Diabetes, Helicobacter pylori, metabolic syndrome

## Abstract

**Aim:**

The aim of this study is to survey the effect of Helicobacter Pylori on metabolic syndrome parameters in diabetic patients.

**Background:**

Helicobacter pylori (*HP*) infection is the most common infection in developing countries. Some studies showed the association between *HP *infection and insulin resistance. Insulin resistance is a major mechanism in the development of metabolic syndrome (MetS) and it is said that MetS is more prevalent among HP infected subjects. Also, some studies have shown that MetS is common among patients with type 2 diabetes mellitus. In this study, we aimed to investigate the prevalence of MetS in diabetic patients and its association with *HP*.

**Methods:**

This cross-sectional study was carried out from May to December 2014 on 211 diabetic patients. For each patient, the following data were collected: age, gender, diabetes duration, weight, body mass index (BMI), waist circumference, blood pressure (BP), HDL, cholesterol, triglyceride (TG), total cholesterol, and HbA1c. The lipid profile was performed on fasting samples. Anti- HP IgG antibody was measured and serum titer >30AU/mL was considered positive. MetS was diagnosed by The National Cholesterol Education Program’s Adult Treatment Panel III report (NCEP-ATPIII) and IDF criteria.

**Results:**

Totally 139 patients (65.9%) were HP+ and 72 patients (34.1%) were HP-. Age, gender and diabetes duration were not significantly different in both groups. BMI was significantly lower in HP+ women (29.05±5.26 vs. 31.45±4.8, p=0.02). Although the waist circumference of men was not different between the two groups but it was significantly lower in HP+ women (102.04±12.37 vs. 97.3±10, p=0.03). Although BP and TG levels were not statistically different in HP+ and HP- patients, but HP+ patients had lower HDL level (p=0.037) which was due to lower HDL in men (58.2±26.6 vs. 72.48±28.1, p=0.012). The prevalence of MetS according to the IDF criteria among HP+ and HP- patients was 76.6% vs. 69.8% (p=0.27). Also, the prevalence of MetS according to NCEP-ATP III criteria among HP+ and HP- patients was 90.4% vs. 87.2% (p=0.5). Duration of diabetes did not affect the prevalence of metabolic syndrome among HP+ and HP- patients.

**Conclusion:**

It seems that HP infection increases the prevalence of metabolic syndrome through an increase in insulin resistance. According to NCEP-ATPIII criteria, the increase in the prevalence of metabolic syndrome in HP+ patients is almost significant, however more complete studies is recommended to investigate this relationship.

## Introduction

Helicobacter pylori (*HP*) infection is the most common infection around the world, particularly in developing countries and its prevalence varies between countries; generally, the prevalence is about 30% in developed and up to 80% in developing countries (1). It is said that *HP *infection plays a role in some endocrine disorders such as autoimmune hyroid diseases, diabetes and primary hyperparathyroidism and may have a high prevalence among patients with diabetes (2,3). The association between *HP *and type 2 diabetes was first explored in Simon et al.’s study (4) and recently, a meta-analysis suggested a trend toward more frequent *HP *infections in diabetic patients (5).

In one study, the rate of *HP *seropositivity among diabetic and non-diabetic patients were 55.8% and 44.2%, respectively and the prevalence of *HP *infection was significantly higher in diabetic patients (6). In addition, some studies have shown an increased incidence of diabetes among *HP+* patients so that the first report that *HP *infection increased incidence of diabetes was in a study by Jeon et al. using a prospective cohort of 782 Latino individuals >60 years of age (7). Etiopathogenesis of *HP *infection in diabetic patients have not been defined clearly. However, this hypothesis is now proposed that *HP *infection is more prevalent among diabetics**. **Some studies examined the association between *HP *infection and insulin resistance (8).

As insulin resistance can develop in the presence of inflammation or as a result of alterations in counter regulatory hormones that affect insulin, *HP *may thus promote insulin resistance by inducing chronic inflammation and affecting insulin-regulating gastrointestinal hormones (8, 9). Another significant point is that the increase in insulin resistance plays a role in developing metabolic syndrome (MetS). The MetS is also known as the insulin resistance syndrome, syndrome X, and the deadly quartet and includes a cluster of heart disease risk factors (10-12). Coronary heart disease, cardiovascular disease, and total mortality are significantly higher in adults with MetS (13). Some studies have shown that MetS is common among patients with type2diabetesandincreasestheriskofcardiovasculardiseaseand all-cause mortality (14, 15). On the other hand, *HP *seropositivity was significantly higher in cases with MetS compared with those without MetS (16). Similar results were reported from Iran in which MetS was found to occur very frequently in the general population, and had a significant association with prior infection with Chlamydia pneumonia, *HP*, cytomegalovirus, and type 1 herpes simplex virus (17)2.

In contrast to non-diabetic populations, few studies have examined the clinical relevance of the new IDF definition on the diagnosis of MetS in type 2 diabetes. This study aimed to investigate the prevalence of MetS among patients with type 2 diabetes and its association with *HP*.

## Materials and Methods

This cross-sectional study was carried out from May to December 2014 on 211 diabetic patients referred to the diabetes clinic of the Shahid Beheshti Hospital of Qom. In case of receiving insulin, pregnancy, smoking, history of *HP *treatment (proton-risk factors: abdominal obesity defined by WC >102cm in men and >88cm in women, serum TG >1.7 mmol/L (150 mg/ dL), HDL cholesterol <1.03mmol/L (<40mg/dL) in men and <1.29 mmol/L (<50mg/dL) in women, blood pressure >130/85 mmHg and a fasting glucose >6.1 mmol/L. Diabetic patients with at least two other risk factors are deemed to have MetS (18).

**IDF**

The new diagnostic criteria are central obesity, defined as WC>94cm and >80cm for Caucasian male and female, respectively or >90cm and >80cm for Asian male and female, respectively; in addition to two of the following: TG>1.7 mmol/L (150mg/dL), or specific treatment for this lipid abnormality; reduced HDL- cholesterol (<1.03mmol/L (<40 mg/dL) in males and <1.29 mmol/L (<50mg/dL) in females) or specific treatment for this lipid abnormality; blood pressure >130/85mmHg; and fasting hyperglycaemia, defined as glucose >5.6 mmol/L (100mg/dL) or previous diagnosis of diabetes or impaired glucose tolerance. T2DM patients with at least one other risk factor after fulfilling the WC threshold are deemed to have MetS (19).

Statistical analysis

Statistical analysis was performed using SPSS software version 16 with either Students t-test or chi-square. P-value<0.05 was considered to be statistically significant. Data are expressed as mean and standard deviation.

Ethics

All individuals signed informed consent prior to their enrollment in the study. Also, the study was planned according to the ethical guidelines following the Declaration of Helsinki and Ethics Committee of Qom University of Medical Sciences.

## Results

In this study 139 patients (65.9%) were HP+ and 72 patients (34.1%) were HP-. Age, gender, weight and diabetes duration were not significantly different in both groups but BMI was significantly lower in HP+ women (29.05±5.26 vs. pump inhibitor, H blocker, and bismuth) or antibiotics in the 31.45±4.8, p=0.02) (table 1). Although the waist circumference previous 6 months, surgery on upper GI tract, gastric cancer and using non-steroidal anti-inflammatory drugs, patients were excluded from the study. For each patient, the following data were collected: age, gender, diabetes duration, weight, body mass index (BMI), waist circumference (WC), blood pressure, HDL, cholesterol, triglyceride (TG), total cholesterol, and HbA1c. The lipid profile was performed on fasting samples. Anti-HP IgG antibody was measured by ELISA kit, made by Padtan Elm Co, Iran and in case of serum titer >30AU/mL, it was considered positive.

MetS was defined using the NCEP-ATP III (National Cholesterol Education Program Adult Treatment Panel III) and IDF (International Diabetes Federation) criteria.

NCEP-ATP III

Diagnosis is based on the presence of three or more of these of men was not different between two groups but it was significantly lower in HP+ women (102.04±12.37 vs. 97.3±10, p=0.03). In terms of other indicators of MetS, although HP+ and HP- groups were not statistically different in blood pressure and TG level, but HP+ patients had lower HDL level (p=0.037) which was due to lower HDL in men (58.2±26.6 vs. 72.48±28.1, p=0.012) (table 1). HP+ patients had higher levels of insulin resistance (4.484±2.781 vs. 3.160±2.327). The prevalence of MetS according to IDF criteria among HP+ and HP- patients was 76.6% vs. 69.8% (p=0.27). Also, the prevalence of MetS according to NCEP- ATP III criteria among HP+ and HP- patients was 90.4% vs. 87.2% (p=0.5). As shown in table 2, duration of diabetes did not affect the prevalence of metabolic syndrome among HP+ and HP- patients.

**Table 1:** Some characteristics of the patients based on HP serology

**Parameter**	**HP+**	**HP-**	**P value**
Age (years)	47.72±5.72	47.06±5.88	0.41
Male (%)	62(49.6)	39(45.3)	0.57
Diabetes duration (years)	8.92±4.5	8.37±4	0.36
Weight (kg)	75.16±11.75	76.93±10.37	0.26
Male	77.05±9.97	77.03±10.3	0.99
Female	73.3±13.1	76.85±10.5	0.13
Body mass index	28.45±4.78	29.74±4.83	0.062
Male	27.85±4.22	27.71±4.08	0.87
Female	29.05±5.26	31.45±4.8	0.02
Waist circumference (cm)	100.78±10.84	101.2±12.05	0.78
Male	104.3±10.58	100.2±11.7	0.07
Female	97.3±10	102.04±12.37	0.03
HbA1c (%)	8.11±1.67	8.08±1.32	0.895
Total cholesterol (mg/dL)	207.3±67.4	205.1±63.2	0.820
HDL (mg/dL)	60.7±26.7	69.2±29.2	0.037
Male	58.2±26.6	72.48±28.1	0.012
Female	61.8±27.6	65.6±28	0.47
Triglyceride (mg/dL)	229.3±114.6	224.2±100.2	0.747
Systolic BP	134.84±8.8	135.75±8.99	0.46
Diastolic BP	83.36±7.28	84.88±7.27	0.15
HOMA-IR	4.484±2.781	3.160±2.327	0.013
Prevalence of MetS			
IDF N(%)	95(76.6%)	60(69.8%)	0.27
NCEP-ATP III N(%)	113(90.4%)	75(87.2%)	0.5

**Table 2:** Prevalence of HP based on diabetes duration

	**IDF (%)**		**NCEP-ATP III (%)**	
**Diabetes duration ** **(year)**	**HP+**	**HP-**	**HP+**	**HP-**
<5	25(73.5%)	20(80.0%)	32(91.4%)	23(92.0%)
5-10	49(80.3%)	27(64.3%)	54(88.5%)	34(81.0%)
10-15	14(77.8%)	9(69.2%)	18(100.0%)	12(92.3%)
15-20	5(62.5%)	4(80.0%)	7(87.5%)	5(100.0%)
>20	2(66.7%)	0(0.0%)	2(66.7%)	1(100.0%)
P value	0.78	0.35	0.376	0.53

## Discussion

Since the metabolic risk factors for type 2 diabetes and cardiovascular disease including abdominal obesity, hyperglycemia, dyslipidemia, and hypertension are common, the existence of a MetS was suggested (20). Other names of MetS are the insulin resistance syndrome, syndrome X, the deadly quartet, or the obesity dyslipidemia syndrome (21). It refers to some risk factors that promote the development of atherosclerotic cardiovascular disease and its clinical role is to identify individuals at risk of this complication. The elevated weight gain is a major risk factor for the MetS. In the Framingham heart study cohort, an increase in weight of ≥2.25kg over 16 years was associated with a 21-45% increase in the risk for developing the syndrome (22).

In addition to age, race, and weight, postmenopausal status, smoking, low income, high carbohydrate diet, no alcohol consumption, and physical inactivity are associated with an increased risk of MetS (23). Chronic subclinical inflammation is increasingly recognized as a part of this syndrome (24). Chronic inflammation and elevated CRP level are the common pathways in the MetS and infectious agents for promotion of the atherosclerotic process. Helicobacter pylori is still the most prevalent infection of the world (17, 25). It is a gram-negative, spiral-shaped pathogenic bacterium that specifically colonizes in the gastric epithelium (26). The infection induces an acute polymorph nuclear infiltration in the gastric mucosa and which is gradually replaced by an immunologically mediated, chronic, predominantly mononuclear cellular infiltrate (27). Also, local production and systemic diffusion of proinflammatory cytokines may exert their effects in remote tissues and organic systems and result in extragastric manifestations (28).

It is said that *HP *infection plays a role in some endocrine disorders (2, 3). In our study, the prevalence of *HP *infection was significantly higher in diabetic patients (p=0.001). In our previous study, the prevalence of *HP *infection was 55.8% in diabetics while it was 44.2% in non-diabetics (6). One of the hypothesis about *HP *infection as a risk factor for diabetes is increased insulin resistance in these patients. One of the first studies in this field was done by Aydemir et al.’s study in 2005 on 63 HP+ and 27 HP- patients. Age, gender, and BMI were not different between both groups. HOMA-IR was 1.73±1.1 in HP- group, whereas it was 2.56±1.54 in HP+ group (8).

In another previous study of us, the HP+ diabetic patients required higher levels of serum insulin to reach the same degree of glycemic control compared to the HP- ones (29). In Patel et al.’s study, according to the IDF and NCEP-ATP III criteria 73.5%, and 89% had MetS, respectively. The prevalence of MetS in non-diabetic subjects was less than this amount. It is estimated that about 20-25% of the world’s population have the MetS (28). In Saudi Arabia, a study of the prevalence of MetS was done in 2005, using the NCEP- ATP III. The overall age-adjusted prevalence was found to be 39.3%. Age-adjusted prevalence in males is 37.2% while females have a higher prevalence of 42% (30). The prevalence of this syndrome is higher and similar to our study in diabetic patients. For example, the prevalence of the MetS in diabetic patients in Nigeria appears to be high (87.1%) (31). In Song et al.’s study IDF vs. NCEP-ATPIII criteria showed high prevalence of MetS (93.1 vs. 90.5%) regardless of gender (IDF: male vs. female, 91.7 vs. 94.8% and NCEP-ATPIII: male vs. female, 87.6 vs. 94.2%) (15). In Tan et al.’s study, the overall prevalence of MetS was 96.1%, and 84.8% using NCEP-ATP III and IDF, respectively (14). In only one study in Ghana, the prevalence of this syndrome among diabetics was low (24%) which may be due to the low waist circumference is these patients (95.99±15.63 cm) (32). In our study, the prevalence of *HP *was 69.5%. *HP *infection may cause dyslipidemia, as it increases total cholesterol, LDL, lipoprotein a, apolopoprotein apo-B, and triglyceride concentration and decreases HDL and the apolipoprotein apoA-1 concentration (33, 34). Eradication of HP in healthy subjects seems to increase HDL and decrease LDL level (33). Also, in our study, HDL level was significantly lower among HP+ individuals (60.7±26.7 vs. 69.2±29.2). We found no association between the prevalence of *HP *infection and MetS in patients with diabetes. No study has examined this association. Some studies have shown this association in non- diabetic population. In a large Japanese population there was a significant and independent association between *HP *and MetS by multiple logistic regression analysis. *HP *seropositivity was significantly associated with higher systolic blood pressure (beta coefficient = 1.03, p=0.014), lower HDL level (beta coefficient =-2.00, p< 0.001), and higher LDL level (beta coefficient= 2.21, p=0.005) (16). In another study, a total of 5889 of healthy Korean adults were included in the analysis. The MetS was more strongly associated with histologic positivity for HP (OR=1.26; 95%CI, 1.08-1.48) than serologic positivity (OR=1.12, 95% CI= 0.95-1.32), after adjusting for age, sex, smoking status, alcohol consumption, and economic status (35). Longo- Mbenza, et al. showed evidence supporting the association of *HP *seropositivity with cardiovascular disease and elevated number of components of MetS (36).

Similar results were reported in Nabipour et al.’s study in which MetS was highly prevalent in the general population, and had a significant association with previous *HP *infection (17). However, there are studies that did not confirm this association. Furthermore, Naja et al.’s findings suggested no association of *HP *infection with insulin resistance or MetS and the prevalence of *HP *infection in was comparable to other developing countries. They concluded that eradication of *HP *infection to prevent insulin resistance or MetS is not warranted (37-39). Anyway, our study showed that despite the significant association between *HP *infection and insulin resistance, no such association was found between MetS and *HP *infection among patients with diabetes. The explanation for this finding can be the high prevalence of both variables in diabetic patients in addition to high levels of HDL in the subjects.

Metabolic syndrome is highly prevalent in Diabetic Patients. About the prevalence of Metabolic syndrome in Diabetic patients, according to IDF criteria, the prevalence of metabolic syndrome in Diabetic Patients with HP+ and HP- was 90.4% vs. 87.2%(p=0.27), and according to NCEP-ATPIII criteria it was 90.4% vs. 87.2%(p=0.5), and the differences was not significant. It seems that HP infection increases the prevalence of metabolic syndrome through an increase in insulin resistance. According to NCEP-ATPIII criteria, the increase in prevalence of metabolic syndrome in HP+ patients is almost significant, however more complete studies is recommended to investigate this relationship.
